# Exercise during pregnancy: knowledge and beliefs of medical practitioners in South Africa: a survey study

**DOI:** 10.1186/s12884-015-0690-1

**Published:** 2015-10-07

**Authors:** Estelle D. Watson, Brydie Oddie, Demitri Constantinou

**Affiliations:** Centre for Exercise Science and Sports Medicine, School of Therapeutic Sciences, Faculty of Health Sciences, University of Witwatersrand, Wits Medical School, Wits, Johannesburg, 2050 South Africa

**Keywords:** Prenatal exercise, Knowledge, Attitudes, Pregnancy, Medical practitioners, Exercise

## Abstract

**Background:**

There is compelling evidence for the benefits of regular exercise during pregnancy, and medical practitioners (MPs) can play an important role in changing antenatal health behaviours. The purpose of this study was to assess the knowledge, attitudes and beliefs of South African MPs towards exercise during pregnancy.

**Methods:**

A convenience sample of ninety-six MPs working in the private health care sector, including General Practitioners (*n* = 58), Obstetricians/Gynaecologists (*n* = 33) and other Specialists (*n* = 5), participated in this cross sectional, descriptive survey study. A 33-item questionnaire was distributed manually at medical practices and via email to an on-line survey tool. Descriptive statistics and frequency tables were calculated for all questions. Chi-squared and Fisher’s exact statistical tests were used to determine the differences in response by age, speciality and years of practice (*p* < 0.05).

**Results:**

The majority of practitioners (98 %) believe that exercise during pregnancy is beneficial, and were knowledgeable on most of the expected benefits. Seventy-eight percent believed that providing exercise advice is an important part of prenatal care, however only 19 % provided informational pamphlets and few (24 %) referred to exercise specialists. A large majority (83 %) were unaware of the recommended exercise guidelines. Although age and years of practice played no role in this awareness, practitioners who focussed on obstetrics and gynaecology were more likely to be aware of the current guidelines, than those in general practice (*p* < 0.001).

**Conclusion:**

Although the MPs were largely positive towards exercise during pregnancy, their advice did not always align with the current guidelines. Therefore, better dissemination of available research is warranted, to bridge the gap between clinical knowledge and current recommendations for physical activity promotion.

**Electronic supplementary material:**

The online version of this article (doi:10.1186/s12884-015-0690-1) contains supplementary material, which is available to authorized users.

## Background

The health benefits of an active pregnancy are well known. Several positive associations between regular activity and maternal outcomes have been clearly demonstrated. For example, an active pregnancy has shown an improvement in cardiovascular and metabolic function, and increased strength and bone density [1]. Regular exercise appears to lower the risk of gestational diabetes mellitus (GDM), gestational hypertension, and preeclampsia [2]. Evidence also exists for the role of exercise in preventing incontinence during pregnancy and in the postpartum period [3, 4]. In addition, it has been shown to reduce excessive gestational weight gain (EGWG), which is an important predictor of numerous adverse maternal outcomes [1].

Even more compelling is the recent evidence for foetal origin of adult disease. Maternal obesity may provide an early embryo environment that appears to have a major impact on the health of the offspring in adulthood [5]. This is particularly important for South African women, where obesity is prevalent and has been found to be strongly associated with physical inactivity [6]. In South Africa (SA), rates of obesity appear to be three times higher in women than in men [7], and 56.5 % of the female population are overweight [6]. Demographic surveys in SA have shown similar gender patterns for inactivity, where 63 % of women are reportedly inactive compared to 48 % of men [8]. Although no data currently exists on the physical activity levels of South African women during pregnancy, literature suggests that women are particularly vulnerable to inactivity, and prescribing exercise during the prenatal period may provide a protective effect against these modifiable risk factors.

As the beneficial evidence for exercise during pregnancy mounts, the American College of Obstetricians and Gynaecologists (ACOG) guidelines have become less restrictive since initially released in 1985 [9]. Unsurprisingly, pregnant women, in the absence of medical or obstetric complications, are encouraged to participate in moderate-intensity aerobic and low-intensity strengthening exercise. Some additional considerations may include restrictions to supine activities, or activities that may increase the risk of falling or contact [4, 9]. Furthermore, exercise duration, frequency and intensity can be prescribed on an individual basis to avoid potential hyperthermia [4]. Although care needs to be taken when prescribing exercise, the benefits of being active during the prenatal period far outweigh the risks [4].

Primary health care providers are well placed to promote exercise to the pregnant population, and may have an important role to play in advising and educating women on healthy behaviours [10;11]. In fact, in the general population, brief counselling from a general practitioner has shown to be a cost effective and successful method of improving activity levels [10]. Therefore, primary care prevention of disease may have a profound effect on, not only the prenatal population, but the health of future generations as well. Although encouraging exercise in women with an uncomplicated pregnancy should form an integral part of prenatal care, little is known about views of SA medical practitioners (MPs) on this subject. Therefore, the purpose of this study was to assess the knowledge, attitudes and beliefs of South African MPs towards exercise during pregnancy.

## Methods

A cross sectional, descriptive survey study was conducted to determine exercise and pregnancy knowledge of MPs. Study participants were a convenience sample of 96 MPs, including General Practitioners (GPs) (*n* = 58), Obstetricians/Gynaecologists (*n* = 33) and other Specialists (*n* = 5). The 33-item questionnaire was designed using published guidelines [11] and adapted from a previous study by Bauer et al. [12]. The questionnaire consisted of 15 likert-type scale questions; 15 selected responses and 3 open-ended questions (see Additional file 1). The questionnaire was piloted for content and construct validity, as well as technical functionality and usability. Questionnaires were distributed: (i) electronically via an emailed hyperlink to the online survey (Using the Survey Monkey online servicehttp://www.surveymonkey.com); and (ii) manually via an appointment at medical practices. Questionnaires were distributed electronically to 958 Obstetricians/Gynaecologists and 562 General Practitioners located in all provinces in South Africa, whilst manual distribution included 100 Obstetricians/Gynaecologists offices within the central Gauteng area. This study used a closed survey recruitment process, whereby the contact details of the selected sample was sourced from the Health Professions Council of South Africa (HPCSA) website (http://www.hpcsa.co.za), or from the internet. In addition, the hyperlink to the survey was circulated by a medical directory service (MEDpages) to their selected distribution list. Medical Practitioners not currently registered with the HPCSA were excluded from the study. In addition, due to time constraints and inaccessibility in public health care clinics, MPs working solely in the public sector were also excluded from the study.

For the reporting of the online survey methodology, the CHERRIES guideline has been followed [13]. An introductory email, explaining the purpose of the study, length of the survey, data collection procedures and other important study details, was sent. Informed consent was provided through selecting the link to the questionnaire at the end of the email. The online survey was set up to include 3–7 items per page and 7 screens in total. All fields were set up as mandatory, which assisted in ensuring completeness of the survey, and respondents were able to go back and review any items at any stage in completion of the questionnaire. To maintain confidentiality, no personal identifying information was collected. The online system was set up in such a way that each participant was provided with a unique link to the webpage, and could therefore not participate in the survey more than once. An online survey completion rate of 0.89 was achieved. Ten participants filled in the questionnaire manually, whilst the remaining 86 participated used the online tool. Participation was voluntary and no incentives for participating were provided. The Human Research Ethics Committee (HREC) of the University of the Witwatersrand approved the study (reference M130445).

Descriptive statistics and frequency tables were calculated for all questions. Chi-squared tests, or Fisher’s exact test, were used to examine the differences in response by (1) age (<40 years or > 40 years); (2) focus of practice (obstetrics/gynaecology or general practice); and (3) years of practice (<15 years or >15 years). All statistical tests were done using Statistica version 11, and statistical significance was set at *p* < 0.05.

## Results

Demographic information for the sample is reported in Table 1. The majority of practitioners were Caucasian (55 %, *n* = 53) and female (64 %, *n* = 61), between the ages of 30–59 (73 %). Thirty two participants had been in practice for over 20 years, and the main focus of practise was obstetrics/gynaecology (46 %).

**Table 1:** Participant demographics (*N* = 96)

*Variable*	*N* (%)
Occupation	
General practitioner	58 (60)
Obstetrician/Gynaecologist	33 (34)
Other Specialist	5 (5)
Gender	
Male	35 (36)
Female	61 (64)
Race	
Caucasian	53 (55)
African	21 (22)
Indian	19 (20)
Coloured	1 (1)
Asian	2 (2)
Age (years)	
<30	6 (6)
30–39	25 (26)
40–49	26 (27)
50–59	19 (20)
>60	20 (21)
Practise location	
Urban	82 (85)
Sub-urban	10 (10)
Rural	4 (4)
Years in practice	
1–5	18 (19)
6–10	15 (16)
11–15	15 (16)
16–20	16 (17)
>20	32 (33)
Main focus of practice	
Obstetrics/Gynaecology	44 (46)
Family Medicine	40 (42)
General practice	12 (13)

Key attitudinal statements of the respondents regarding exercise during pregnancy is shown in Table 2 and these were largely positive. For example, 98 % of respondents believe that exercise is beneficial during pregnancy. The majority of responders (78 %) believed that exercise promotion is an important component of prenatal care, and 74 % felt that this advice influenced patient behaviour. Most MPs (94 %) recommended that their patients participate in moderate exercise during pregnancy; however there appears to be a misalignment between the published recommendations and clinical practice. For example, 18 % reported that previously sedentary women should not embark on an exercise programme during pregnancy, and 42 % did not recommend a strength-training programme.

**Table 2:** Attitudes of practitioners to key questions (*N* = 96)

Statement	Numbers (%) of responders who indicated			
	Strongly agree	Agree	Disagree	Strongly disagree
Exercising during pregnancy is beneficial	64 (67)	30 (31)	2 (2)	0 (0)
Advising patients on exercise during pregnancy is not a major component of prenatal care	4 (4)	17 (18)	41(43)	34 (35)
Pregnant patients follow the advice given during their office visits	7 (7)	64 (67)	23 (24)	2 (2)
A sedentary woman, with an uncomplicated pregnancy, should not begin an exercise programme during pregnancy	6 (6)	11 (12)	58 (60)	21 (22)
Pregnant women who are chronic exercisers should be encouraged to continue an exercise programme throughout pregnancy	42 (44)	47 (49)	7 (7)	0 (0)
Pregnant women should not participate in a strength-training programme during pregnancy	12 (13)	28 (29)	47 (49)	9 (9)
During pregnancy, women should be recommended to exercise at a moderate intensity	31 (32)	59 (62)	6 (6)	0 (0)
Exercise during pregnancy increases the risk of low birth weight babies	4 (4)	7 (7)	48 (50)	37 (39)
The possible harmful effects of exercise on the foetus are minimal if not non-existent	26 (27)	57 (59)	7 (7)	6 (6)

**Fig. 1:**
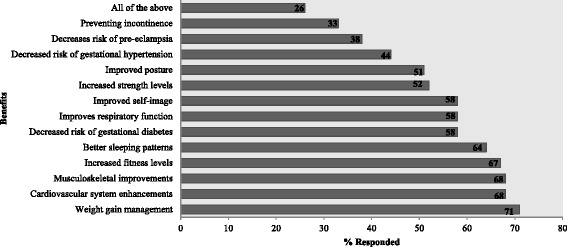
Benefits of exercise during pregnancy as indicated by respondents (*N* = 96)

The perceived benefits of exercise during pregnancy are shown in Fig. 1. The majority of practitioners believed in the benefits of exercise for weight management (71 %) as well as cardiovascular (68 %) and musculoskeletal (68 %) health. Few were convinced of the effects of exercise on blood pressure response (44 %) and pelvic floor strength (33 %).

Statements regarding exercise prescription knowledge and practises are shown in Table 3. Over half the respondents (56 %) reported that patients enquired about exercise during a visit. Despite this demand from patients, few (19 %) practitioners provide any written advice or informational pamphlets, and only 18 % provide individualised exercise prescription. Furthermore, 69 % do not routinely provide exercise restrictions and 15 % believe that low intensity exercise is sufficient to gain health benefits. A large majority (83 %) of the respondents were not familiar with the ACOG guidelines for exercise during pregnancy; however, an encouraging 71 % of practitioners reported they would be interested in attending a continuous professional development (CPD) workshop on the subject. When assessing prenatal referral practices, approximately a quarter (24 %) of the respondents reported that they did not refer their patients to other healthcare providers for exercise. In fact, 46 % reported they were unaware of any exercise classes or trainers in their area. On the other hand, 39 % reported that they would refer to a Biokineticist (Clinical Exercise Specialist), 26 % to a personal trainer, and 21 % to a Physiotherapist.

**Table 3:** Responses of participants to statements regarding exercise prescription during pregnancy

Statement	Numbers of responders % (n)			
	(*n* = 85)			
	Never	Seldom	Often	Always
Do your pregnant patients ask you questions about exercise during pregnancy?	7 (8)	31 (37)	44 (52)	3 (4)
Do you provide informational pamphlets on pregnancy and exercise to your patients?	43 (51)	26 (31)	13 (15)	3 (4)
Do you obtain exercise histories on your pregnant patients?	10 (12)	27 (32)	34 (40)	14 (17)
Do you give each pregnant patient an individualised exercise programme for her to follow?	39 (46)	28 (33)	15 (18)	3 (4)
	Very aware	Aware	Vaguely aware	Unaware
Are you aware of the 2002 ACOG guidelines for exercise during pregnancy	6 (5)	21 (18)	29 (25)	44 (37)
	Yes	No		
Would someone from your practice be interested in attending a workshop on pregnancy and exercise? (*n* = 82)	71 (58)	29 (24)		
Do you routinely give exercise restrictions to your pregnant patients	31 (26)	69 (59)		
Are you aware of any exercise classes or trainers in your area? (*n* = 82)	54 (44)	46 (38)		
	Low	Moderate	Vigorous	
What intensity would you recommend your patients exercise at? (*n* = 82)	15 (12)	83 (68)	2 (2)	
	Biokineticist	Personal trainer	Physiotherapist	Other
Who do you refer your patients to for exercise prescription?	39 (33)	26 (22)	21 (18)	14 (12)

Approximately 72 % of the younger respondents offered advice on exercise, and although this appeared to decrease with age (69 % in >40 years), the difference was not significant (*p* = 0.730). Practitioners whose practices focussed on obstetrics and gynaecology were more likely to be aware of the current guidelines than those in general practice (*p* < 0.001). However, awareness of the guidelines did not have a statistically significant effect on beliefs regarding the benefits of exercise during pregnancy (*p* = 0.124), nor on strength training (*p* = 0.117) or intensity (*p* = 0.583) recommendations. In addition, age (*p* = 0.374) and years of practice (*p* = 0.248) played no role in awareness of the ACOG guidelines.

## Discussion

There is considerable evidence for the positive effects of exercise during a healthy pregnancy [1], and this belief appears to be strongly supported by MPs, in this, and other similar studies [12, 14]. Exercise is a powerful tool to manage excessive gestational weight gain, and its associated complications [2], a view which is well supported in the current study. In addition, our study found that over half the respondents were knowledgeable on the role of exercise in improving cardiovascular fitness, strength, sleeping patterns and reducing the risk of gestational diabetes. However, the findings suggest that most practitioners were unaware of the benefits of exercise in preventing and treating incontinence. Women are encouraged to initiate pelvic floor exercises during pregnancy, and in the postpartum period, a concept that should be reinforced at a primary healthcare level [3, 4]. Likewise, a recent systematic review suggests a reduced risk of preeclampsia with exercise [15], but few of the practitioners in this study were aware of this. Although the MPs in this study had a good knowledge of most of the benefits of exercise during pregnancy, awareness and education of its role in preventing incontinence and preeclampsia needs to be improved.

There appears to be a common disconnect between research and clinical knowledge on exercise during pregnancy. Other studies have highlighted the lack of healthcare provider’s knowledge of ACOG guidelines [12, 16, 17]. Similarly, the authors found that only a small portion of the practitioners in this study were aware of the ACOG guidelines. In fact, locally the South African Sports Medicine Association recently released its own position statement on prenatal exercise prescription [4], encouraging practitioners to advise on moderate exercise, inclusive of strength training, to all healthy pregnant patients. This statement, along with the ACOG guidelines, provides a clear outline for exercise prescription and applicable restrictions. However, the lack of awareness of the guidelines may explain why many of the practitioners in this study did not routinely provide restrictions, and many believed that one should not participate in strength-training during pregnancy. Furthermore, despite the health risks of being sedentary during pregnancy [4, 18], there is still a portion of practitioners that are reluctant to encourage previously sedentary patients to embark on an exercise programme during pregnancy. Nonetheless, when compared to their overseas counterparts, many more SA practitioners appear to encourage these pregnant women to start a new exercise programme [16, 17].

Studies have suggested that pregnancy may be viewed by clinicians as a high-risk state, in which even marginal foetal risk should be avoided, despite the potential benefit to the mother [19]. In line with this, many practitioners in this study believed women should exercise at low intensities. This is consistent with a study by Evenson et al.[16], where most obstetricians were inclined to recommend mild over moderate exercise. Thus, there is a need for paradigm shift in how activity during pregnancy is viewed, as well as improved accuracy of the advice given. Healthcare providers should be reminded that in the absence of pathology or health risk to mother and/or foetus, pregnancy is a physiological state with benefits to be gained from exercise. There appears to be a great need for better dissemination of current guidelines and research to healthcare practitioners.

Encouragingly, the majority of the study participants indicated their willingness to attend a workshop on the subject. This interest in continued education has been echoed in other studies [12, 16], and can provide great potential for improving the occurrence and impact of physician advice, which can have an integral role to play in improving activity levels at a primary healthcare level . In fact, a study by Lewis and Lynch [20] found that physician training can double the amount of advice given. Therefore, training in the form of workshops may be warranted. This education and evidence-based medicine should be furthermore incorporated into the undergraduate medical degree curriculum.

Research has shown that healthcare providers can have a positive effect on their patient’s attitudes towards exercise [2, 21]. Indeed, gaining knowledge of the benefits of exercise may motivate pregnant women to become more active [1]. Our study showed that over half the women asked their providers about exercise. Similarly, a study by de Jersey et al. [22] reported that women are interested in receiving education, but there appears to be a disparity between what they want and what is provided by their healthcare practitioners. Prenatal advice available to women is often overwhelming, and various healthcare providers should provide an accurate and standard message [22]. Vague and conflicting information may explain why women tend to reduce their activity levels during pregnancy [23], making correct exercise prescription in the prenatal period all the more important.

### Study strengths and limitations

Although similar studies have been done in other countries [13;17;18], this study is the first of its kind assessing South African healthcare providers beliefs and attitudes towards exercise prescription during pregnancy. The South African guideline for exercise during pregnancy is available [4], however such research is essential to determine if, and how, these public health messages are being provided at a primary care level. Furthermore, MPs may play a crucial role in implementation of interventions for this vulnerable population and therefore their attitudes towards exercise prescription, and delivery, provides useful formative research in the field. The study does, however, have certain limitations. Due to known low response rates from MPs, together with the convenience sampling design, these results may not be reflective of SA MPs as a whole. Additionally, as with similar studies [17], response bias may be a limitation due to non-randomisation of the sample, and the potential for selective bias (those who agreed to participate were interested in the topic). This bias may overestimate the results and further research should expand to reach a wider population, with more reliable sampling techniques. In addition, the exclusion of practitioners working in the public sector eliminates knowledge of practice that may affect the vast majority of the South African population. Lastly, a qualitative research design may provide useful insights into counselling barriers and facilitators, which will better inform future intervention strategies.

## Conclusion and implications for practice

The SA medical profession appears to largely support the belief that exercise is beneficial during pregnancy, and believe that they have a role to play in exercise promotion on a primary care level, but lacked accurate specifics with regard to exercise prescription (mode, frequency, intensity and duration) and are not adequately promoting it to their patients. As research on prenatal exercise has increased in the past two decades, it has become imperative that innovative strategies are put in place to ensure the gap between research and clinical practise is bridged. There appears to be a great need for provision of clear, evidence-based information through continuous education activities that can be shared with patients. Improving maternal health is a key directive of the World Health Organisation (WHO), and MPs can have major influence in promoting exercise, and changing health behaviours, in pregnant women.

## Additional file

Additional file 1:
**Questionnaire.docx.** (DOCX 75 kb)
