# Supplementary material for: Exercise during pregnancy: knowledge and beliefs of medical practitioners in South Africa: a survey study
Source: BMC Pregnancy Childbirth. 2015 Oct 7;15:245. doi: 10.1186/s12884-015-0690-1 (PMC4597379; doi:10.1186/s12884-015-0690-1)
Supplement: Additional file 1: — Questionnaire.docx. (DOCX 75 kb) [file 12884_2015_690_MOESM1_ESM.docx]

**Additional file 1: Questionnaire**

| ID Code |  |
| --- | --- |

**Please tick the response that most closely ﬁts your demographics. If the choices given do not ﬁt your situation, please feel free to write in one that does**.

1. What is your current occupation?

🞎G.P. 🞎 Obstetrician 🞎 Gynaecologist 🞎 Specialist Other _____________

2. Where is your practice located?

🞎 Urban 🞎 Sub-urban area Other____________

3. How many years have you been practicing?

🞎 1-5 🞎 6-10 🞎 11-15 🞎16-20 🞎>20

4. What is the main focus or specialty of your practice?

🞎 Obstetrics 🞎Gynaecology 🞎 Family medicine Other_____________

5. What is your gender?

🞎 Male 🞎 Female

6. What is your race?

🞎 Caucasian 🞎African 🞎Asian 🞎Indian 🞎Coloured Other____________

7. What races make up 20% or more of your patient population? (Please tick all that apply.)

🞎Caucasian 🞎African 🞎Asian 🞎Indian 🞎Coloured Other____________

8. What is your age?

🞎<30 🞎30-39 🞎 40-49 🞎 50-59 🞎>60

9. What percent of your practice involves obstetrics?

🞎 100% 🞎75% 🞎50% 🞎25% Other_____________

**Please tick the response that most closely ﬁts your feelings toward the statement given. If examples are requested, please write all those that you feel apply in the space provided.**

10. Exercising during pregnancy is beneﬁcial.

🞎 Strongly agree 🞎Agree 🞎 Disagree 🞎 Strongly disagree

11. Advising patients on exercise during pregnancy is not a major component of prenatal care.

🞎 Strongly agree 🞎Agree 🞎 Disagree 🞎 Strongly disagree

12. If exercise is discussed by you or your staff, it does not include individualized recommendations.

🞎 Strongly agree 🞎Agree 🞎 Disagree 🞎 Strongly disagree

13. Pregnant patients follow the advice given during their ofﬁce visits.

🞎 Strongly agree 🞎Agree 🞎 Disagree 🞎 Strongly disagree

14. A sedentary woman, with an uncomplicated pregnancy, should not begin an exercise program during pregnancy.

🞎 Strongly agree 🞎Agree 🞎 Disagree 🞎 Strongly disagree

15. Pregnant women who are chronic exercisers should be encouraged to continue an exercise program throughout pregnancy.

🞎 Strongly agree 🞎Agree 🞎 Disagree 🞎 Strongly disagree

16. Pregnant women should not participate in a strength-training program during pregnancy.

🞎 Strongly agree 🞎Agree 🞎 Disagree 🞎 Strongly disagree

17. What do you feel are the main beneﬁts of exercising during pregnancy?

- Postural changes
- Increased fitness levels
- Increased strength levels
- Improved self-image
- Better sleeping patterns
- Increased viability of the placenta
- Increased amniotic fluid
- Preventing incontinence
- Decreased risk of gestational diabetes
- Decreases risk of pre-eclampsia
- Decreased risk of gestational hypertension
- Cardiovascular system enhancements
- Musculoskeletal improvements
- Endocrine system changes
- Improves respiratory function
- Weight gain management

18. During pregnancy, woman should be recommended to exercise at moderate intensity.

🞎 Strongly agree 🞎Agree 🞎 Disagree 🞎 Strongly disagree

19. Exercising during pregnancy increases the risk of low birth weight babies.

🞎 Strongly agree 🞎Agree 🞎 Disagree 🞎 Strongly disagree

20. The possible harmful effects of exercise on the fetus are minimal if not nonexistent.

🞎 Strongly agree 🞎Agree 🞎 Disagree 🞎 Strongly disagree

**Please tick the answer that most clearly represents what you do in your practice. When yes and no choices are given, if you answer yes, please continue and answer the lettered questions related to the initial question. If you answer no, please continue on to the next numbered question. If examples are requested, please write all that you feel apply in the space provided.**

21. Does your ofﬁce give advice to your pregnant patients about pregnancy and exercise?

🞎 Yes 🞎 No

If no, please skip to question 22.

a. If yes, who in your ofﬁce gives this advice?

🞎 Yourself 🞎Nurse Other____________

b. If yes, at what stage in pregnancy would this occur? (Tick all that apply.)

🞎 Initial visit 🞎1^st^ trimester 🞎2^nd^ trimester 🞎3^rd^ trimester 🞎Postpartum

c. How long would a typical exercise advice session take?

🞎 5-10 minutes 🞎11-20 minutes 🞎21-30 minutes 🞎>30 minutes

22. Do your pregnant patients ask you questions about exercising during pregnancy?

🞎 Never 🞎 Seldom 🞎 Often 🞎 Always

23. Do you provide informational pamphlets on pregnancy and exercise to your patients?

🞎 Never 🞎 Seldom 🞎 Often 🞎 Always

24. Do you obtain exercise histories on your pregnant patients?

🞎 Never 🞎 Seldom 🞎 Often 🞎 Always

25. Do you give each pregnant patient an individualized exercise program for her to follow?

🞎 Never 🞎 Seldom 🞎 Often 🞎 Always

26. Are you aware of the 2002 ACOG guidelines for pregnancy and exercise?

🞎Very aware 🞎Aware 🞎Vaguely aware 🞎Unaware

27. Do you routinely give exercise restrictions to your pregnant patients?

🞎 Yes 🞎 No

If no, please skip to question 28.

a. If yes, please list some of the examples below:

28. Who do you refer your pregnant patients to for exercise recommendations?

🞎 Personal trainer 🞎Biokinetics 🞎Physiotherapist Other _____________

29. Do you recommend your patients exercise during pregnancy?

🞎 Yes 🞎 No

If no, please skip to question 29.

a. If yes, what types of exercise do you recommend for your patients? (Please circle all that apply; feel free to add others.)

🞎 Walking 🞎Running 🞎Swimming 🞎Cycling 🞎Aerobics

Other

30. Do you recommend your patients avoid certain types of exercise?

🞎 Yes 🞎 No

If no, please skip to question 30.

a. If yes, please write examples in the space below:

31. Are you aware of any exercise classes or trainers in your area that could beneﬁt your patients?

🞎 Yes 🞎 No

If no, please skip to question 31.

a. If yes, do you recommend your patients go to any of these opportunities?

🞎 Never 🞎 Seldom 🞎 Often 🞎 Always

32. Would you or someone from your practice be interested in attending a workshop on pregnancy and exercise if offered?

🞎 Yes 🞎 No

a. If no, please explain in space below:

33. What intensity would you recommend your patients exercise at?

🞎 Low 🞎 Moderate 🞎 Vigorous
